# Can women empowerment boost dietary diversity among children aged 6–23 months in sub-Saharan Africa?

**DOI:** 10.1186/s41182-024-00579-3

**Published:** 2024-06-04

**Authors:** Richard Gyan Aboagye, Irene Esi Donkoh, Joshua Okyere, Abdul-Aziz Seidu, Bright Opoku Ahinkorah, Sanni Yaya

**Affiliations:** 1 Department of Family and Community Health, Fred N. Binka School of Public Health, University of Health and Allied Sciences, Hohoe, Ghana; 2 Department of Medical Laboratory Science, University of Cape Coast, Cape Coast, Ghana; 3 Department of Population and Health, University of Cape Coast, Cape Coast, Ghana; 4 School of Nursing and Midwifery, College of Health Sciences, Kwame Nkrumah University of Science and Technology, Kumasi, Ghana; 5 Centre for Gender and Advocacy, Takoradi Technical University, Takoradi, Ghana; 6 College of Public Health, Medical and Veterinary Sciences, James Cook University, Townsville, Australia; 7 School of Public Health, Faculty of Health, University of Technology Sydney, Sydney, Australia; 8 School of International Development and Global Studies, University of Ottawa, 120 University Private, Ottawa, K1N 6N5 Canada; 9 The George Institute for Global Health, Imperial College London, London, UK

**Keywords:** Dietary diversity, Child health, SWPER, Women's empowerment, Women’s health

## Abstract

**Background:**

The empowerment of women has implications on the health and dietary needs of children. Using the survey-based women’s empowerment index (SWPER), we examined the association between women’s empowerment and dietary diversity among children aged 6–23 months in sub-Saharan Africa.

**Methods:**

Data from the Demographic and Health Surveys of 21 countries were utilized. Descriptive spatial map was used to present the proportions of dietary diversity among the children. Multilevel binary logistic regression was used to examine the association between SWPER and dietary diversity.

**Results:**

Overall, 22.35% of children aged 6–23 months had adequate minimum dietary diversity (MDD) in sub-Saharan Africa. The countries with the highest proportions of adequate MDD were Angola, Benin, Madagascar, Rwanda, Sierra Leone, and South Africa. South Africa had the highest proportion of MDD (61.00%), while Liberia reported the least (9.12%). Children born to mothers who had high social independence were more likely to have adequate MDD compared to those with low social independence [aOR = 1.31, 95% CI 1.21, 1.41]. In addition, children born to women with medium [aOR = 1.12; 95% CI 1.03, 1.21] and high decision-making [aOR = 1.25, 95% CI 1.14, 1.37] were more likely to receive MDD than those with low decision-making.

**Conclusions:**

Insufficient dietary diversity is evident among children aged 6–23 months in sub-Saharan Africa. MDD in children is influenced by women's empowerment. Policies and interventions promoting women's empowerment can enhance MDD, especially for vulnerable groups in rural and poorer households. It is crucial to leverage media and poverty reduction strategies to improve MDD among children in sub-Saharan African countries.

**Supplementary Information:**

The online version contains supplementary material available at 10.1186/s41182-024-00579-3.

## Background

Infant and young child feeding (IYCF) practices are of enormous public health concern. Positively, adequate variety of diets with the right amount of nutrients are essential for the growth and development of children and mitigate the emergence of dietary and nutritional disorders [1, 2]. In the absence of good diet, children become susceptible to nutritional problems and other infectious diseases [1]. This has led to the urgent calls for the adequate provision of breastfeeding and complementary feeding to infants and young children [1]. One of such advocated strategies is the provision of adequate minimum dietary diversity (MDD). MDD refers to the consumption of at least five out of the eight food groups, which include breast milk, grains, roots and tubers, legumes and nuts, dairy products, flesh foods (such as meat, fish, poultry, and organ meats), eggs, vitamin A-rich fruits and vegetables, and other fruits and vegetables [1].

Dietary diversity has long been recognized as a reliable predictor of dietary quality and as an indicator of micronutrient deficiencies in the diet of children [1]. Specifically, as countries face economic crises, children globally continue to suffer from undernutrition, overweight/obesity, and micronutrient deficiencies [2]. Thus, making the nutritional status of infants and young children a global public health concern. Due to children's rapid growth and development, this age group needs meals with a high nutrient density and variety. As such, dietary diversity assessment aids in determining whether the child’s diet has the critical nutrients required for growth. Consuming a range of foods boosts micronutrient sufficiency, which is essential for children’s healthy development and nutrition [3]. Although nutrition-specific initiatives such as the promotion of IYCF practices have demonstrated success in addressing children’s malnourishment [4], this is not rapidly helping due to the limited financial support to facilitate a wider scale. However, women’s empowerment has been considered as a necessary intervention for enhancing children’s nutrition [5].

The concept of women’s empowerment has varying interpretations with numerous definitions by many; however, the main motive is to give women social, economic, and political power [6, 7]. Women can only attain empowerment if they can envision other alternative life forms and believe that they are capable of and entitled to making decisions [8]. The achievement of gender equality and women's empowerment was identified as the fifth Sustainable Development Goal (SDG) in 2015. This goal is crucial for advancing sustainable development [9]. It can be accomplished by consciously and intentionally empowering women and girls [10]. This height when attained could contribute to  women's development and enrich human resources to achieve gender equality and ultimately the SDG 3 and 5 [10].

The empowerment of women in health and socioeconomic status influences child growth but it is complicated and multifaceted, making it exceedingly challenging to quantify due to its abstract and comprehensive nature [11, 12]. Hitherto, the lack of agreement on how to quantify women’s empowerment, particularly in the absence of global standard indicators, has prevented accountability in low- and middle-income countries (LMICs). This has resulted in the paucity of empirical studies to support the progress of women’s empowerment and dietary diversity in LMICS. Although some indicators such as the Gender Gap Index, Gender Development Index, and the Gender Inequality Index have been proposed [13, 14], yet, they have deficiencies such as concentrating on data from a region to represent a country and are biased towards a group of women [15].

Notwithstanding, the first individual-level indicator to allow comparisons between several countries over time was established and accredited in 2017 using the Demographic and Health Survey (DHS) data from 34 African nations: the survey-based women’s empowerment index (SWPER), which identifies three categories of empowerment that reflect partnered women (married or in a union) resources and agency [12]. The categories encompass social independence (mostly made up of the prerequisites that help women realize their goals in education, access to information, significant life events, and marital assets), decision making (represents the level of the woman’s engagement in decision-making), and attitudes to violence (a proxy for a woman’s incorporation of gender norms-related acceptability of violence) [12, 16]. The SWPER categories correspond to enabling conditions, instrumental agency, and intrinsic agency. All of these categories allow women to develop power, make choices, and have conscious aspirations, respectively [17].

Although the SWPER is aimed at improving childcare and reducing poverty to promote the health and nutrition of women and children, most surveys have failed to include this aspect. This is particularly true in LMICs like some countries in sub-Saharan Africa (SSA), where both women's empowerment and dietary diversity are significant issues [18]. A study conducted in Ethiopia indicates that SWPER indicators are associated with reducing child nutritional deficiency [19]. However, it is unknown how this change in SWPER over time has affected the child's dietary diversity [20].

Evidence from an earlier report in 2021 revealed that 24% of infants and young children (aged 6–23 months) in Eastern and Southern Africa did not meet the minimum requirements for five out of the eight recommended dietary groups [21]. While the issue of child and maternal nutrition continues to be a public health concern, and SWPER has evolved, it is important to consider whether all dimensions of SWPER could impact dietary diversity in children aged 6–23 months [22]. Therefore, this study examined the association between SWPER indicators and MDD among children aged 6–23 months in SSA.

## Methods

### Data source

This study was conducted in SSA with data sourced from the DHS of 21 countries who had datasets from 2015 to 2021. The countries and their respective data used can be accessed via https://dhsprogram.com/data/available-datasets.cfm. The detailed DHS methodology has been highlighted in the literature [23, 24]. Briefly, the DHS is a nationwide survey conducted in over 90 LMICs to ascertain health and demographic trends [23]. A cross-sectional design was adopted for the survey. A two-stage cluster sampling method was used to recruit the respondents for the survey [24, 25]. Pretested structured questionnaires were used to collect data from the respondents on several health indicators, including IYCF practices and women’s empowerment. DHS program used trained data collectors to carry out the data collection. We included a weighted sample of 54,750 mother–child pairs in our study. We followed the Strengthening the Reporting of Observational Studies in Epidemiology (STROBE) guidelines in writing this paper [26] (Table 1).

**Table 1:** Description of study sample per country

Country	Year of survey	Weighted sample	Weighted percentage
1. Angola	2015–16	3018	5.51
2. Benin	2017–18	2959	5.40
3. Burundi	2016–17	2982	5.45
4. Cameroon	2018	2288	4.18
5. Ethiopia	2016	2437	4.45
6. Gambia	2019–20	1612	2.94
7. Guinea	2018	1694	3.09
8. Liberia	2019–20	1072	1.96
9. Madagascar	2021	2737	5.00
10. Mali	2018	2291	4.18
11. Malawi	2015–16	3793	6.93
12. Mauritania	2019–2021	2503	4.57
13. Nigeria	2018	7475	13.65
14. Rwanda	2019–20	1822	3.33
15. Sierra Leone	2019	2120	3.87
16. Chad	2014–15	4060	7.41
17. Tanzania	2015–16	2180	3.98
18. Uganda	2016	3303	6.03
19. South Africa	2016	817	1.49
20. Zambia	2018	2163	3.95
21. Zimbabwe	2015	1424	2.60
All countries	2015–2021	54,750	100.00

### Variables

MDD was the outcome variable. The World Health Organization (WHO) stipulated that children aged 6–23 months should be fed with breastmilk and at least four of these seven food groups: grains, roots, and tubers; legumes and nuts; dairy products (milk, yogurt, cheese); flesh foods (meat, fish, poultry, liver, or other organs); eggs; vitamin A-rich fruits and vegetables; and other fruits and vegetables [1]. Hence, children aged 6–23 months who were fed with at least five food groups were coded as ‘1 = yes’, indicating that the child obtained an adequate MDD, otherwise coded ‘0 = no’ [1, 27].

SWPER was the key explanatory variable in our study. SWPER is a globally accepted indicator of women's empowerment developed for use in LMICs [12]. Ewerling et al. [12] posit that the SWPER is a comprehensive indicator tested and validated for within-country and between-country comparisons. In the same study, the authors further stipulated that SWPER can be used as an outcome or a determinant of health. The detailed variables used and their categorisation have been provided in the literature [12, 16, 28]. SWPER was developed using fifteen variables initially [12]. However, it was later revised to include only fourteen variables: beating not justified if wife goes out without telling husband, beating not justified if wife neglects the children, beating not justified if wife argues with husband, beating not justified if wife refuses to have sex with husband, beating not justified if wife burns the food, frequency of reading newspaper or magazine, woman education, age of respondent at cohabitation, age of respondent at first birth, age difference: woman’s minus husband’s age, education difference: woman’s minus husband’s years of schooling, who usually decides on respondent’s health care, who usually decides on large household purchases, and who usually decides on visits to family or relatives [16] (Table 2). The detailed coding, equations, and comprehensive description of the variables are available elsewhere [12, 16, 28]. These 14 variables were used to develop the three domains of women’s empowerment, namely, attitude to violence, social independence, and decision-making [16, 28]. Social independence or autonomy denotes the preconditions, such as the schooling attainment, information access, age at crucial life events, and spousal asset differentials that allow women to realize their goals. Decision-making on the other hand refers to the degree of the woman’s involvement in household decisions which can also be viewed as a gauge of instrumental agency. Finally, attitude to violence closely relates to the concept of intrinsic agency and is a proxy for the woman’s incorporation of gender norms—related to the acceptability of intimate partner violence [1]. In our study, each domain was categorised into low, medium, and high. We followed Baye et al.'s coding of SWPER in our study [22]. The distribution of the dimensions of SWPER per country have been provided in Additional file 1: Table S1, attached to this paper.

**Table 2:** Summary of items used in each domain of SWPER

Item
Attitude to violence
1. Beating justified if wife goes out without telling husband
2. Beating justified if wife neglects the children
3. Beating justified if wife argues with husband
4. Beating justified if wife refuses to have sex with husband
5. Beating justified if wife burns the food
Social independence
6. Frequency of reading newspaper or magazine
7. Woman education in completed years of schooling
8. Age of woman at first birth (this was computed for women who had not had a child as well)
9. Age at first cohabitation
10. Age difference: woman’s minus husband’s age
11. Education difference: woman’s minus husband’s years of schooling
Decision-making
12. Who usually decides on respondent’s health care
13. Who usually decides on large household purchases
14. Who usually decides on visits to family or relatives

We included 10 variables as covariates. Two criteria were used to select these covariates. First, the covariates had a significant association with dietary diversity from literature [22, 29]. Second, the variables were available in the DHS datasets. The covariates consisted of sex of child, age of child, birth order, antenatal care visits, place of delivery, postnatal care attendance, size of household, household wealth index, place of residence, and geographical sub-region. The covariates were further segregated into individual and contextual level based on literature [27, 29].

### Statistical analyses

Our analysis was carried out in four stages. First, spatial map was used to present the results of the proportions of adequate MDD among children aged 6–23 months. In the second stage, we examined the distribution of adequate MDD across SWPER by cross-tabulation and showed the distribution of the dimensions of SWPER across the countries (Additional file 1). Pearson chi-square test of independence was used to determine the variables significantly associated with MDD. We used multilevel binary logistic regression analysis to examine the association between SWPER and MDD, controlling for the covariates. Model O was the empty model and it denotes the variance in MDD attributed to the primary sampling unit (PSU) with no key explanatory variable or covariates. Model I contained the domains of SWPER. Model II was fitted to contain the domains of SWPER and the individual-level covariates. Model III contained the domains of SWPER and the contextual-level covariates. Model IV was fitted to contain the domains of SWPER and all the covariates. All five models had fixed and random results. The random results denoted the measure of variation in the MDD based on PSU [measured by Intra-Class Correlation Coefficient (ICC)], whereas fixed results denoted the association between the explanatory variable and/or covariates and the MDD. The results of the fixed effect model were presented using adjusted odds ratio (aOR) with their respective 95% confidence interval (CI). All the analyses were weighted per the DHS guidelines [23]. Statistical significance was set at *p* < 0.05. For the random effect results, the Akaike’s Information Criterion (AIC) and log likelihood values were used to select the best-fitted model based on the smallest AIC and highest log likelihood values. Model IV was chosen as the best-fitted model, since it had the least AIC and the highest log likelihood values. We used Stata version 17.0 to perform all the analyses.

### Estimation

The equations representing the multilevel binary logistic regression models are as follows:

- ${Y}_{ij}$ denotes the binary outcome variable (MDD) for individual *i* in cluster (or level-2 unit) *j.*
- ${{\text{AV}}}_{ij} ,{{\text{SI}}}_{ij} ,{{\text{DM}}}_{ij}$ are the three domains of SWPER (attitude to violence, social independence, and decision-making, respectively) for individual *i* in cluster *j.*
- ${{\text{Covariates}}}_{ij}$ as a vector of covariates (sex of child, age of child, birth order, antenatal care visits, place of delivery, postnatal care attendance, size of household, household wealth index, place of residence, geographical sub-region) for individual *i* in cluster *j*.
  $${\text{log}}it\left( {\frac{{P\left( {Y_{ij} = 1} \right)}}{{1 - P\left( {Y_{ij} = 1} \right)}}} \right) = \beta o_{j} + u_{j} + e_{ij}$$

Model with no explanatory variable

$${\text{log}}it\left( {\frac{{P\left( {Y_{ij} = 1} \right)}}{{1 - P\left( {Y_{ij} = 1} \right)}}} \right) = \beta 0 + \beta_{{{\text{AV}}}} {\text{AV}}_{ij} + \beta_{{{\text{SI}}}} {\text{SI}}_{ij} + \beta_{{{\text{DM}}}} {\text{DM}}_{ij} + u_{j} + e_{ij}$$

Model with the SWPER domains

$${\text{log}}it\left( {\frac{{P\left( {Y_{ij} = 1} \right)}}{{1 - P\left( {Y_{ij} = 1} \right)}}} \right) = \beta 0 + \beta_{{{\text{AV}}}} {\text{AV}}_{ij} + \beta_{{{\text{SI}}}} {\text{SI}}_{ij} + \beta_{{{\text{DM}}}} {\text{DM}}_{ij} + \beta_{{{\text{COV}}}} {\text{Covariates}}_{ij} + u_{j} + e_{ij}$$

Model with the SWPER domains and individual level covariates (sex of child, age of child, birth order, antenatal care visits, place of delivery, and postnatal care attendance).

$${\text{log}}it\left( {\frac{{P\left( {Y_{ij} = 1} \right)}}{{1 - P\left( {Y_{ij} = 1} \right)}}} \right) = \beta 0 + \beta_{{{\text{AV}}}} {\text{AV}}_{ij} + \beta_{{{\text{SI}}}} {\text{SI}}_{ij} + \beta_{{{\text{DM}}}} {\text{DM}}_{ij} + \beta_{{{\text{COV}}2}} {\text{Covariates}}_{ij2} + u_{j} + e_{ij}$$

Model with the SWPER domains and contextual-level covariates (size of household, household wealth index, place of residence, and geographical sub-region).

$${\text{log}}it\left( {\frac{{P\left( {Y_{ij} = 1} \right)}}{{1 - P\left( {Y_{ij} = 1} \right)}}} \right) = \beta 0 + \beta_{{{\text{AV}}}} AV_{ij} + \beta_{{{\text{SI}}}} {\text{SI}}_{ij} + \beta_{{{\text{DM}}}} {\text{DM}}_{ij} + \beta_{{{\text{COV}}}} {\text{Covariates}}_{ij} + \beta_{{{\text{COV}}2}} {\text{Covariates}}_{ij2} + u_{j} + e_{ij}$$

Model with domains of SWPER and all the covariates.

### Ethical consideration

Ethical clearance was not sought for this study because the DHS datasets are freely available for use. However, before using the dataset for publication, we obtained permission from the Monitoring and Evaluation to Assess and Use Results Demographic and Health Surveys (MEASURE DHS). The detailed ethical guidelines are available at http://goo.gl/ny8T6X.

## Results

### Proportions of minimum dietary diversity across the 21 countries in sub-Saharan Africa

**Fig. 1:**
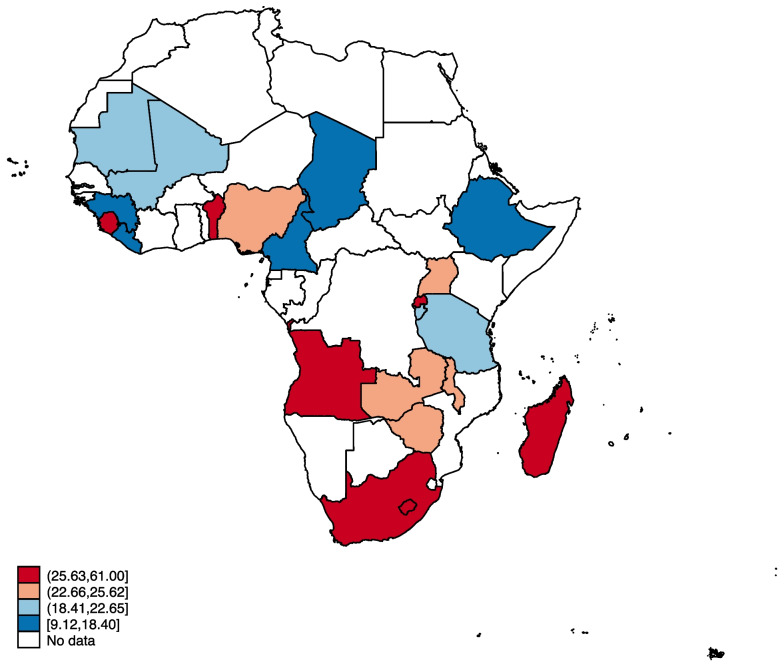
Proportions of minimum dietary diversity across the 21 countries in sub-Saharan Africa

The study shows that 22.35% of children aged 6–23 in SSA had MDD. The hotspot countries for MDD were Angola, Benin, Madagascar, Rwanda, Sierra Leone, and South Africa. South Africa had the highest proportion of MDD (61.00%), while Liberia (9.12%) reported the least (Fig. 1).

### Distribution of minimum dietary diversity across the explanatory variables

Table 3 presents the distribution of MDD across the various explanatory variables. Women who scored high in all three SWPER domains had the highest proportions of adequate MDD. These domains are high attitude to violence (24.4%), high social dependence (30.7%), and high decision-making (27.2%). Both male (22.3%) and female (22.4%) children had similar proportions of adequate MDD. Higher proportions of MDD were observed among children aged 12–17 (25.9%) and among firstborns (24.7%).

**Table 3:** Distribution of minimum dietary diversity across the explanatory variables

Variable	Weighted		Minimum dietary diversity	
	Frequency	Percentage	Adequate % [95% CI]	*p* value
Attitude to violence				< 0.001
Low	15,739	28.7	18.4 [17.5, 19.3]	
Medium	9853	18.0	22.5 [21.4, 23.6]	
High	29,158	53.3	24.4 [23.7, 25.2]	
Social independence (autonomy)				< 0.001
Low	18,218	33.3	17.1 [16.3, 17.9]	
Medium	19,805	36.2	20.1 [19.4, 20.9]	
High	16,727	30.5	30.7 [29.7, 31.8]	
Decision-making				< 0.001
Low	13,078	23.9	17.5 [16.6, 18.5]	
Medium	26,341	48.1	21.9 [21.2, 22.7]	
High	15,331	28.0	27.2 [26.1, 28.3]	
Sex of child				0.811
Male	27,916	51.0	22.3 [21.6, 23.0]	
Female	26,834	49.0	22.4 [21.7, 23.1]	
Age of child (in months)				< 0.001
6–8	10,053	18.4	11.9 [11.1, 12.8]	
9–11	9214	16.8	20.7 [19.6, 21.8]	
12–17	19,649	35.9	25.9 [25.0, 26.8]	
18–23	15,834	28.9	25.6 [24.6, 26.5]	
Birth order				< 0.001
1	9771	17.8	24.7 [23.5, 25.9]	
2–4	27,050	49.4	23.8 [23.0, 24.5]	
5 and above	17,929	32.8	18.9 [18.1, 19.7]	
Number of antenatal care visits				< 0.001
None	6959	12.7	14.9 [13.7, 16.2]	
1–3	17,046	31.1	20.2 [19.4, 21.0]	
4 or more	30,745	56.2	25.2 [24.5, 26.0]	
Place of delivery				< 0.001
Home	19,002	34.7	16.0 [15.2, 16.8]	
Health facility	35,029	64.0	25.8 [25.1, 26.5]	
Other	719	1.3	23.5 [19.2, 28.4]	
Postnatal care attendance				< 0.001
No	38,326	70.0	19.9 [19.3, 20.5]	
Yes	16,424	30.0	28.1 [27.1, 29.2]	
Household size				< 0.001
Small	24,604	44.9	23.7 [22.9,24.5]	
Medium	23,706	43.3	21.4 [20.6,22.1]	
Large	6440	11.8	20.8 [19.5,22.2]	
Wealth index				< 0.001
Poorest	12,604	23.0	14.7 [13.8, 15.6]	
Poorer	12,275	22.4	17.8 [16.9, 18.7]	
Middle	11,119	20.3	20.4 [19.4, 21.4]	
Richer	9852	18.0	26.4 [25.2, 27.6]	
Richest	8900	16.3	37.4 [35.9, 39.0]	
Place of residence				< 0.001
Urban	16,156	29.5	31.3 [30.1, 32.5]	
Rural	38,594	70.5	18.6 [18.0, 19.2]	
Geographical sub-regions				< 0.001
Central Africa	9366	17.1	18.6 [17.0, 20.3]	
Southern Africa	4404	8.0	31.1 [28.9, 33.4]	
Eastern Africa	19,255	35.2	23.1 [22.2, 24.0]	
Western Africa	21,725	39.7	21.6 [20.7, 22.4]	

*p* values were generated from Chi-square test

Women who had four or more antenatal care attendance (25.2%), those who delivered at the health facility (25.8%), and women who attended postnatal care (28.1%) reported high proportions of MDD. In addition, higher proportion of adequate MDD was observed among children in small households (23.75), those in households with richest wealth index (37.4%), among those residing in urban areas (31.3%), and those in Southern SSA (31.1%).

### Association between SWPER and minimum dietary diversity

Table 4 shows the association between SWPER and MDD in SSA. Children born to mothers who had high social independence [aOR = 1.31, 95% CI 1.21, 1.41] were more likely to receive adequate MDD compared to those with low social independence. In addition, children born to women with medium [aOR = 1.12; 95% CI 1.03, 1.21] and high decision-making [aOR = 1.25, 95% CI 1.14, 1.37] were more likely receive MDD than those with low decision-making. In terms of the covariates, higher odds of MDD was observed among children aged 9–11 months [aOR = 1.94, 95% CI 1.74, 2.16], 12–17 months [aOR = 2.75, 95% CI 2.50, 3.04], and 18–23 months [aOR = 2.64, 95% CI 2.41, 2.90] compared to younger children (6–8 months). We observed higher likelihood of MDD among children born in health facilities [aOR = 1.25, 95% CI 1.15, 1.35] and those whose mothers attended postnatal care [aOR = 1.37, 95% CI 1.27, 1.47] compared to those who were born at home and those whose mothers did not attend postnatal care, respectively. Also, the odds of receiving MDD increased with increasing wealth index, with the highest likelihood among children from the richest household [aOR = 2.42, 95% CI 2.15, 2.72]. Rural-dwelling women’s children were less likely [aOR = 0.79, 95% CI 0.72, 0.87] to receive MDD compared to those in urban areas.

**Table 4:** Association between the dimensions of SWPER and minimum dietary diversity

Variable	Model O	Model I aOR [95% CI]	Model II aOR [95% CI]	Model III aOR [95% CI]	Model IV aOR [95% CI]
Fixed effect model					
Attitude to violence					
Low		1.00	1.00	1.00	1.00
Medium		1.18*** [1.09, 1.28]	1.13* [1.04, 1.23]	1.09* [1.00, 1.19]	1.07 [0.98, 1.17]
High		1.23*** [1.14, 1.32]	1.16*** [1.08, 1.24]	1.09* [1.02, 1.17]	1.07 [0.99, 1.15]
Social independence (autonomy)					
Low		1.00	1.00	1.00	1.00
Medium		1.17*** [1.10, 1.25]	1.06 [0.99, 1.14]	1.06 [0.99, 1.13]	1.02 [0.95, 1.09]
High		1.90*** [1.77, 2.05]	1.61*** [1.49, 1.72]	1.39*** [1.29, 1.50]	1.31*** [1.21, 1.41]
Decision-making					
Low		1.00	1.00	1.00	1.00
Medium		1.21*** [1.13, 1.31]	1.14** [1.05, 1.23]	1.16*** [1.07, 1.25]	1.12* [1.03, 1.21]
High		1.48*** [1.36, 1.62]	1.35*** [1.24, 1.47]	1.30*** [1.19, 1.42]	1.25*** [1.14, 1.37]
Age of child (in months)					
6–8			1.00		1.00
9–11			1.92*** [1.73, 2.14]		1.94*** [1.74, 2.16]
12–17			2.68*** [2.44, 2.95]		2.75*** [2.50, 3.04]
18–23			2.59*** [2.36, 2.83]		2.64*** [2.41, 2.90]
Birth order					
1			1.00		1.00
2–4			1.01 [0.94, 1.09]		1.00 [0.92, 1.08]
5 and above			0.93 [0.85, 1.01]		0.93 [0.85, 1.02]
Number of antenatal care visits					
None			1.00		1.00
1–3			1.05 [0.94, 1.18]		1.01 [0.90, 1.14]
4 or more			1.18 [1.06, 1.32]		1.08 [0.96, 1.20]
Place of delivery					
Home			1.00		1.00
Health facility			1.45*** [1.37, 1.60]		1.25*** [1.15, 1.35]
Other			1.27 [0.96, 1.68]		1.23 [0.93, 1.63]
Postnatal care attendance					
No			1.00		1.00
Yes			1.41*** [1.32, 1.50]		1.37*** [1.27, 1.47]
Household size					
Small				1.00	1.00
Medium				1.00 [0.95, 1.07]	1.05 [0.99, 1.13]
Large				1.05 [0.96, 1.16]	1.10 [1.00, 1.21]
Wealth index					
Poorest				1.00	1.00
Poorer				1.26*** [1.16, 1.37]	1.24*** [1.14, 1.35]
Middle				1.43*** [1.31, 1.57]	1.39*** [1.27, 1.53]
Richer				1.80*** [1.63, 2.00]	1.75*** [1.57, 1.94]
Richest				2.53*** [2.25, 2.85]	2.42*** [2.15, 2.72]
Place of residence					
Urban				1.00	1.00
Rural				0.76*** [0.70, 0.84]	0.79*** [0.72, 0.87]
Geographical subregions					
Central Africa				1.00	1.00
Southern Africa				1.73*** [1.49, 2.01]	1.34*** [1.14, 1.57]
Eastern Africa				1.30*** [1.15, 1.46]	1.20** [1.07, 1.36]
Western Africa				1.18** [1.04, 1.33]	1.08 [0.96, 1.22]
Random effect model					
PSU variance (95% CI)	0.78 [0.65, 0.93]	0.68 [0.57, 0.83]	0.71 [0.59, 0.86]	0.70 [0.58, 0.84]	0.72 [0.60, 0.88]
ICC	0.191	0.172	0.178	0.175	0.181
Wald Chi-square	Reference	534.17***	1190.73***	954.88***	1535.06***
Model fitness					
Log-likelihood	− 129,082.89	− 126,684.34	− 123,187.45	− 124,019.85	− 121,198.47
AIC	258,169.8	253,384.7	246,410.9	248,075.7	242,452.9
*N*	54,750	54,750	54,750	54,750	54,750
Number of clusters	1373	1373	1373	1373	1373

*aOR* adjusted odds ratios, *CI* Confidence Interval, * *p* < 0.05, ** *p* < 0.01, *** *p* < 0.001; 1.00 = Reference category; *PSU* Primary Sampling Unit, *ICC* Intra-Class Correlation Coefficient, *AIC* Akaike’s Information Criterion

### Association between SWPER and minimum dietary diversity segregated by sub-regions in sub-Saharan Africa

Table 5 presents the results of the association between SWPER indicators and MDD per geographical sub-region. Medium [aOR = 1.27, 95% CI 1.10, 1.71] and high [aOR= 1.40, 95% CI 1.17, 1.68] attitude to violence were positively associated with MDD only in Central Africa whereas high attitude to violence was associated with MDD in Southern Africa [aOR = 1.41, 95% CI 1.10, 1.80]. In Southern Africa, children born to women with high social independence were more likely to receive MDD compared to those with low social independence [aOR = 1.99, 95% CI 1.48, 2.67]. For Eastern Africa, the likelihood of children receiving MDD was higher among women with medium [aOR = 1.33, 95% CI 1.18, 1.51] and high [aOR = 1.86, 95% CI 1.64, 2.11] social independence relative to those in the low category. Children born to mothers with medium [aOR = 1.39, 95% CI 1.15, 1.69] and high [aOR = 1.61, 95% CI 1.31, 1.98] decision-making in Central Africa were more likely to receive MDD compared to their counterparts with low decision-making. Similarly, children whose mothers had medium [aOR = 1.20, 95% CI 1.04, 1.39] and high [aOR = 1.46, 95% CI 1.25, 1.70] decision-making were more likely to receive MDD compared to those whose mothers had low decision-making. In Western Africa, children whose mothers had high social independence were more likely to receive MDD compared to their counterparts whose mothers belonged to the low category [aOR = 1.12, 95% CI 1.01, 1.24].

**Table 5:** Association between SWPER and minimum dietary diversity segregated by sub-regions in sub-Saharan Africa

Variable	Central Africa aOR [95% CI]	Southern Africa aOR [95% CI]	Eastern Africa aOR [95% CI]	Western Africa aOR [95% CI]
Attitude to violence				
Low	1.00	1.00	1.00	1.00
Medium	1.27* [1.10, 1.71]	1.19 [0.88, 1.62]	1.13 [0.99, 1.28]	0.93 [0.83, 1.05]
High	1.40** [1.17, 1.68]	1.41** [1.10, 1.80]	1.10 [0.99, 1.22]	0.92 [0.84, 1.00]
Social independence (autonomy)				
Low	1.00	1.00	1.00	1.00
Medium	0.90 [0.75, 1.07]	1.08 [0.80, 1.45]	1.33*** [1.18, 1.51]	0.96 [0.88, 1.06]
High	0.85 [0.69, 1.06]	1.99*** [1.48, 2.67]	1.86*** [1.64, 2.11]	1.12* [1.01, 1.24]
Decision-making				
Low	1.00	1.00	1.00	1.00
Medium	1.39** [1.15, 1.69]	1.31 [0.88, 1.94]	1.20* [1.04, 1.39]	1.05 [0.96, 1.15]
High	1.61*** [1.31, 1.98]	1.22 [0.83, 1.81]	1.46*** [1.25, 1.70]	1.05 [0.94, 1.18]
Age of child (in months)				
6–8	1.00	1.00	1.00	1.00
9–11	1.52** [1.14, 2.01]	1.82** [1.23, 2.68]	1.79*** [1.54, 2.09]	2.34*** [2.00, 2.74]
12–17	2.14*** [1.69, 2.72]	2.66*** [1.90, 3.71]	2.27*** [1.98, 2.59]	3.62*** [3.16, 4.16]
18–23	2.32*** [1.81, 2.97]	2.56*** [1.82, 3.61]	1.99*** [1.73, 2.28]	3.58*** [3.11, 4.12]
Birth order				
1	1.00	1.00	1.00	1.00
2–4	0.98 [0.78, 1.22]	0.99 [0.76, 1.28]	0.94 [0.84, 1.05]	1.09 [0.97, 1.22]
5 and above	1.02 [0.79, 1.31]	0.91 [0.64, 1.28]	0.81** [0.70, 0.94]	1.06 [0.93, 1.20]
Number of antenatal care visits				
None	1.00	1.00	1.00	1.00
1–3	0.82 [0.65, 1.04]	1.16 [0.53, 2.50]	1.26 [0.98, 1.62]	0.98 [0.85, 1.12]
4 or more	0.93 [0.75, 1.15]	1.31 [0.61, 2.82]	1.29* [1.01, 1.67]	1.10 [0.96, 1.26]
Place of delivery				
Home	1.00	1.00	1.00	1.00
Health facility	1.15 [0.95, 1.39]	1.34 [0.94, 1.91]	1.18** [1.05, 1.32]	1.19*** [1.08, 1.32]
Other	0.86 [0.27, 2.79]	0.88 [0.39, 1.97]	0.95 [0.70, 1.31]	2.39** [1.58, 3.63]
Postnatal care attendance				
No	1.00	1.00	1.00	1.00
Yes	1.20 [1.00, 1.44]	1.19 [0.93, 1.52]	1.24** [1.13, 1.36]	1.47*** [1.36, 1.60]
Household size				
Small	1.00	1.00	1.00	1.00
Medium	0.92 [0.77, 1.12]	1.27* [1.01, 1.60]	1.07 [0.96, 1.19]	1.03 [0.94, 1.13]
Large	0.84 [0.65, 1.10]	0.81 [0.52, 1.26]	0.95 [0.76, 1.20]	1.13* [1.01, 1.26]
Wealth index				
Poorest	1.00	1.00	1.00	1.00
Poorer	1.10 [0.86, 1.40]	1.36* [1.02, 1.80]	1.34*** [1.17, 1.54]	1.11 [0.98, 1.24]
Middle	1.17 [0.91, 1.49]	1.17 [0.86, 1.57]	1.49*** [1.30, 1.72]	1.29*** [1.15, 1.46]
Richer	1.78*** [1.36, 2.32]	0.93 [0.62, 1.39]	1.92*** [1.67, 2.21]	1.56*** [1.36, 1.79]
Richest	1.61** [1.19, 2.17]	1.08 [0.68, 1.71]	3.46*** [2.96, 4.05]	2.18*** [1.87, 2.54]
Place of residence				
Urban	1.00	1.00	1.00	1.00
Rural	0.51*** [0.42, 0.62]	0.38*** [0.27, 0.53]	0.97 [0.85, 1.10]	0.99 [0.90, 1.10]

* *p* < 0.05, ** *p* < 0.01, *** *p* < 0.001

## Discussion

The first 1000 days of a child is recognised as a window of opportunity to improve their nutritional status [18]. Consequently, there has been an increasing interest in issues relating to how adequately children meet the MDD. To contribute to the wide body of scholarship on the subject, we examined the association between women’s empowerment (using the SWPER) and MDD in children aged 6–23 months in SSA. Only 22.35% of children had adequate MDD. The proportion of MDD in our study is similar to the findings of a previous study conducted in SSA (25.1%) [29]. It is possible that the low levels of MDD in SSA may be influenced by various factors such as food insecurity, poverty, and other maternal and contextual factors. Cultural beliefs regarding food and feeding practices of children may have also played a role in the low MDD found in our study. This low MDD for children underscores the urgency for governments in the respective sub-Saharan African countries to prioritize and invest heavily in addressing existing barriers that limit the potential of children to achieve adequate dietary diversity.

At the country level, the significant difference in MDD between South Africa (highest at 61.00%) and Liberia (lowest at 9.12%) can be attributed to factors related to dietary habits, food availability, and socioeconomic conditions in these two countries [30]. South Africa’s higher prevalence of MDD may be due to its relatively more diverse and developed food supply chain, as well as better access to a variety of foods in urban areas. In addition, South Africa’s population may have greater awareness of dietary diversity and its importance for nutrition, which can lead to improved dietary choices. On the other hand, Liberia's lower prevalence of MDD could be attributed to challenges in accessing food, limited economic resources, and a higher rate of food insecurity [30]. Traditional dietary practices and cultural preferences may also contribute to the limited variety of foods consumed in Liberia.

Our study confirms the hypothesis that women’s empowerment significantly predicts the MDD of children in SSA. Evidence from this study suggests that medium and high social independence and decision-making were associated with a higher likelihood of MDD in children. However, attitudes towards violence was not significant. Similar findings have been reported in previous studies [22, 31] that showed that autonomy (social independence) and decision-making were the only empowerment indicators that predicted children’s MDD. Women with medium and high autonomy and decision-making may have more control over resources, such as income and assets within their households. This control offers them an opportunity to allocate resources for food, nutrition, and health-related needs of the family, including diverse and nutritious food choices. In SSA, children’s feeding practices are usually influenced by other individuals besides the mother (e.g., grandparents, siblings, etc.) [32]. These people often hold certain beliefs and dietary practices that undermine the attainment of MDD. However, women who scored high in autonomy and decision-making are more likely to understand the importance of a diverse diet for optimal health and nutrition, go against widely held beliefs, and thus make informed choices regarding food selection and preparation [22]. Thus, our study emphasizes a need for sub-Saharan African countries to integrate women’s empowerment in their interventions and programmes rolled out to improve the nutritional of children including dietary diversity. We also observed that SWPER scores predicted MDD differently for the sub-regions. While attitude towards violence significantly predicted MDD only in Central Africa, social independence was associated with MDD in Southern and Eastern Africa. SWPER decision-making also predicted MDD only in Central and Eastern Africa. Further studies are needed to fully understand these associations.

### Policy implications

The positive association between SWPER and MDD highlights the importance of promoting and supporting women’s empowerment initiatives to enhance the MDD of children. Policymakers and governments should prioritize the allocation of resources towards comprehensive women’s empowerment programs that focus on enhancing educational opportunities, economic participation, and decision-making capabilities. There is a need to leverage the media and postnatal care attendance as avenues to educate and raise mothers’ awareness about the importance of practicing adequate dietary diversity for their children. Our findings suggest that investing in reducing household poverty would have a trickling effect on mothers’ SWPER index, thereby influencing them to practice adequate dietary diversity. The findings that high social independence and decision-making increase the likelihood of meeting the MDD, make it imperative for policymakers in SSA to invest in empowerment programs offering education, vocational training, and resource access to enhance women’s independence. Strengthening legal frameworks promoting gender equality and women’s rights, such as property, marital, and inheritance rights, is essential. Moreover, nutrition programs must adopt gender-sensitive strategies, empowering women to make informed dietary choices and engage in income-generating activities.

### Strengths and limitations

The SWPER is the most reliable and validated individual-level measurement tool for measuring women's empowerment. In addition, the use of the DHS provides large nationally representative data that allows us to extrapolate our findings to the SSA population. The study was, however, not without limitations. Using the SWPER index limits us to only partnered/married women. This means that our findings are not generalizable to women who are not in any union. In addition, key cultural norms and beliefs and health literacy variables could not be accounted for due to the use of secondary data. Hence, the inferences drawn from this study should be based on the available variables. In addition, MDD was assessed using qualitative measures which makes it prone to biases.

## Conclusion

Less than half of children aged 6–23 months receive adequate dietary diversity. Our study has shown that SWPER significantly predicts the dietary diversity of children in SSA. Therefore, integrating women’s empowerment in policies, programmes, and interventions aimed at improving MDD for children would yield more effective results. Such programmes should target high-risk populations, including children born into poorer households and those in rural areas. The study concludes that the media can be used to promote sufficient dietary diversity for children. It is also important to prioritize household poverty reduction strategies to further enhance dietary diversity for children in SSA.

### Supplementary Information

**Additional file 1: Table S1.** Distribution of dimensions of SWPER index across the countries.

## Data Availability

Data for this study were sourced from Demographic and Health surveys (DHS) and available here: http://dhsprogram.com/data/available-datasets.cfm.
