# Supplementary material for: Can women empowerment boost dietary diversity among children aged 6–23 months in sub-Saharan Africa?
Source: Trop Med Health. 2024 Jun 4;52:39. doi: 10.1186/s41182-024-00579-3 (PMC11149264; doi:10.1186/s41182-024-00579-3)
Supplement: Supplementary file 1 — Additional file 1: Table S1. Distribution of dimensions of SWPER index across the countries. [file 41182_2024_579_MOESM1_ESM.docx]

**Table S1: Distribution of dimensions of SWPER index across the countries**

| **Country** | **Decision-making** | | | **Autonomy** | | | **Attitude to violence** | | |
| --- | --- | --- | --- | --- | --- | --- | --- | --- | --- |
|  | **Low** | **Medium** | **High** | **Low** | **Medium** | **High** | **Low** | **Medium** | **High** |
| 1. Angola | 5.86 | 46.73 | 47.40 | 31.80 | 38.28 | 29.92 | 14.45 | 15.62 | 69.93 |
| 1. Benin | 30.07 | 54.51 | 15.41 | 35.12 | 40.28 | 24.60 | 20.41 | 14.11 | 65.48 |
| 1. Burundi | 13.67 | 67.06 | 19.27 | 16.94 | 49.19 | 33.87 | 34.42 | 25.84 | 39.74 |
| 1. Cameroon | 35.48 | 41.19 | 23.32 | 35.08 | 33.85 | 31.07 | 15.88 | 15.61 | 68.51 |
| 1. Ethiopia | 9.87 | 67.87 | 22.26 | 46.37 | 35.68 | 17.95 | 48.52 | 20.23 | 31.25 |
| 1. Gambia | 33.93 | 52.97 | 13.10 | 33.97 | 34.03 | 32.00 | 32.93 | 23.16 | 43.91 |
| 1. Guinea | 37.45 | 47.85 | 14.71 | 50.74 | 31.19 | 18.07 | 57.83 | 17.11 | 25.06 |
| 1. Liberia | 12.59 | 44.66 | 42.76 | 31.84 | 38.70 | 29.46 | 23.27 | 16.99 | 59.73 |
| 1. Madagascar | 2.00 | 44.36 | 53.65 | 32.37 | 37.94 | 29.69 | 11.17 | 29.88 | 58.95 |
| 1. Mali | 63.52 | 28.31 | 8.17 | 48.47 | 35.84 | 15.69 | 58.38 | 23.91 | 17.71 |
| 1. Malawi | 14.75 | 53.36 | 31.88 | 22.93 | 48.45 | 28.62 | 5.61 | 10.76 | 83.63 |
| 1. Mauritania | 23.71 | 51.64 | 24.64 | 36.13 | 32.17 | 31.69 | 16.23 | 13.57 | 70.20 |
| 1. Nigeria | 39.02 | 39.99 | 21.00 | 41.93 | 23.75 | 34.31 | 23.98 | 8.66 | 67.36 |
| 1. Rwanda | 6.71 | 61.46 | 31.84 | 3.64 | 28.41 | 67.95 | 30.50 | 17.17 | 52.33 |
| 1. Sierra Leone | 42.98 | 41.79 | 15.23 | 38.15 | 38.17 | 23.68 | 40.23 | 15.98 | 43.79 |
| 1. Chad | 38.59 | 43.43 | 17.99 | 60.77 | 29.10 | 10.14 | 57.09 | 21.66 | 21.25 |
| 1. Tanzania | 15.29 | 58.73 | 25.98 | 18.97 | 40.15 | 40.88 | 41.52 | 21.73 | 36.75 |
| 1. Uganda | 12.81 | 44.89 | 42.29 | 22.80 | 44.29 | 32.91 | 24.50 | 26.46 | 49.04 |
| 1. South Africa | 1.58 | 37.63 | 60.79 | 2.47 | 13.60 | 83.94 | 1.00 | 6.75 | 92.25 |
| 1. Zambia | 10.47 | 46.44 | 43.09 | 20.83 | 48.67 | 30.50 | 30.55 | 18.68 | 50.77 |
| 1. Zimbabwe | 4.23 | 39.72 | 56.05 | 10.45 | 39.74 | 49.80 | 14.23 | 28.07 | 57.70 |
